# Supplementary material for: Whole Genome Sequencing and CRISPR/Cas9 Gene Editing of Enterotoxigenic Escherichia coli BE311 for Fluorescence Labeling and Enterotoxin Analyses
Source: Int J Mol Sci. 2022 Jul 6;23(14):7502. doi: 10.3390/ijms23147502 (PMC9321511; doi:10.3390/ijms23147502)
Supplement: Supplementary file 1 [file ijms-23-07502-s001.zip › ijms-1768364-supplementary.pdf]

Supplementary Table S1. Predicted virulence factors of BE311

	BE311 genome	O78:H11:K80 str. H10407 (NC_017633)	UMNK88 (NC_017641)	Details
**Adhesion**	afaA	-	-	Afimbrial adhesin AFA-I
	ecpA	+	+	E. coli common pilus (ECP) A
	ecpB	+	+	E. coli common pilus (ECP) B
	ecpC	+	+	E. coli common pilus (ECP) C
	ecpD	+	+	E. coli common pilus (ECP) D
	ecpE	+	+	E. coli common pilus (ECP) E
	ecpR	+	+	E. coli common pilus (ECP) R
	elfA	+	+	E.coli laminin-binding fimbriae (ELF) A
	elfC	+	+	E.coli laminin-binding fimbriae (ELF) C
	elfD	+	+	E.coli laminin-binding fimbriae (ELF) D
	elfG	+	+	E.coli laminin-binding fimbriae (ELF) G
	eaeH	+	+	
	hcpA	+	+	Hemorrhagic E.coli pilus (HCP) A
	hcpB	+	+	Hemorrhagic E.coli pilus (HCP) B
	hcpC	+	+	Hemorrhagic E.coli pilus (HCP) C
	faeC	-	-	K88 fimbriae
	faeD	-	-	K88 fimbriae
	faeE	-	-	K88 fimbriae

	faeF	-	-	K88 fimbriae
	faeH	-	-	K88 fimbriae
	faeI	-	-	K88 fimbriae
	faeJ	-	-	K88 fimbriae
	fimA	+	-	Type I fimbriae
	fimC	+	-	Type I fimbriae
	fimD	+	-	Type I fimbriae
	fimE	+	-	Type I fimbriae
	fimF	+	-	Type I fimbriae
	fimG	+	+	Type I fimbriae
	fimH	+	-	Type I fimbriae
	fimI	+	-	Type I fimbriae
**Autotransporter**	agn43	-	-	Antigen 43
	cah	+	+	
	ehaB	-	+	
**Invasion**	ibeB	+	+	Invasion of brain endothelial cells (Ibes)
	ibeC	+	+	Invasion of brain endothelial cells (Ibes)
	tia	+	-	Tia/Hek
**Toxin**	Hemolysin/cytoly sin A	+	+	Hemolysin/cytolysin A

Supplementary Table S2. Gene islands of BE311

GI_ID	seq_ID	start	end	GI_length
GI1	assembly_1	788352	882952	94601
GI2	assembly_1	994763	1015054	20292
GI3	assembly_1	1865171	1890949	25779
GI4	assembly_1	1910140	1945302	35163
GI5	assembly_1	3055696	3084439	28744
GI6	assembly_1	3238539	3249070	10532
GI7	assembly_1	3371156	3400521	29366
GI8	assembly_1	3420734	3447970	27237
GI9	assembly_1	3690565	3742184	51620
GI10	assembly_1	3795242	3812772	17531
GI11	assembly_1	4124838	4133341	8504
GI12	assembly_1	4161389	4194337	32949
GI13	assembly_1	4214111	4225195	11085
GI14	assembly_2	18262	26938	8677

Supplementary Table S3. Predicted CRISPR sequences of BE311

ID	source	type	start	end	score	sequence
gnl|Prokka|CRISPR1	minced:0.4.2	repeat_region	1098184	1098577	7	GTGTTCCCCGCGCCAGCGGGGATAAACC
gnl|Prokka|CRISPR2	minced:0.4.2	repeat_region	1124128	1124829	12	GAGTTCCCCGCGCCAGCGGGGATAAACCG

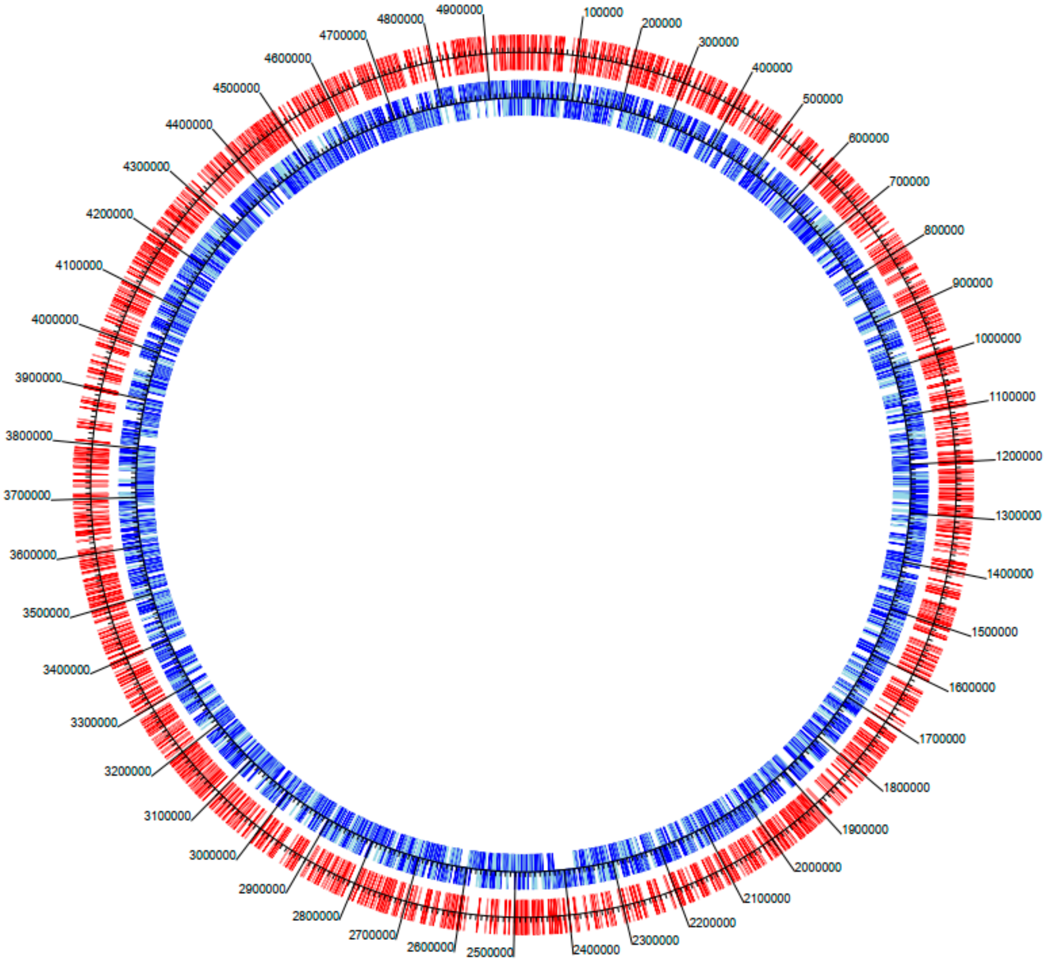

Supplementary Figure S1. Predicted pseudogenes of BE311
